# A Photoresponsive Smart Covalent Organic Framework**

**DOI:** 10.1002/anie.201503902

**Published:** 2015-06-10

**Authors:** Ning Huang, Xuesong Ding, Jangbae Kim, Hyotcherl Ihee, Donglin Jiang

**Affiliations:** Department of Materials Molecular Science, Institute for Molecular Science, National Institutes of Natural Sciences5-1 Higashiyama, Myodaiji, Okazaki 444-8787 (Japan); Center for Nanomaterials and Chemical Reactions, Institute for Basic Science, KAISTDaejeon 305-701 (Republic of Korea)

**Keywords:** anthracene, covalent organic frameworks, photoresponsive materials, porous polymer, smart materials

## Abstract

Ordered π-columnar structures found in covalent organic frameworks (COFs) render them attractive as smart materials. However, external-stimuli-responsive COFs have not been explored. Here we report the design and synthesis of a photoresponsive COF with anthracene units as the photoresponsive π-building blocks. The COF is switchable upon photoirradiation to yield a concavo-convex polygon skeleton through the interlayer [4π+4π] cycloaddition of anthracene units stacked in the π-columns. This cycloaddition reaction is thermally reversible; heating resets the anthracene layers and regenerates the COF. These external-stimuli-induced structural transformations are accompanied by profound changes in properties, including gas adsorption, π-electronic function, and luminescence. The results suggest that COFs are useful for designing smart porous materials with properties that are controllable by external stimuli.

Materials with structures that are transformable in response to external stimuli, such as light, heat and pressure, are attracting increasing attention because of their broad applications in various fields. In particular, when the structural transformations are accompanied by changes in physicochemical properties, these materials are considered “smart” and “dynamic” and can function as stimuli-responsive materials. Two-dimensional covalent organic polymers (2D COFs) and their layered covalent organic frameworks (COFs) are a class of crystalline porous polymers that allow atomically precise integration of organic units into periodic columnar π-arrays and ordered one-dimensional (1D) open channels.[1–7] The π-units in 2D COFs not only constitute π-columns that control the electronic functions but also shape the channel walls that form the interface for the adsorption of gases.[2–4] The integration of stimuli-responsive π-units into COFs is likely to yield structurally dynamic frameworks in which the structure can be transformed upon external stimulation. However, a “smart” COF is unprecedented and the possibility of structural transformation is to be exemplified.

Herein, for the first time, we report a photo-responsive, structurally dynamic COF. The photo-responsive 2D COF (Figure 1 a, Ph-An-COF) was synthesized by condensation of 2,3,6,7-tetrahydroxyanthracene and 1,3,5-benzenetriboronic acid under solvothermal conditions in 92 % isolated yield (Supporting Information, Tables S1–S4; Figures S1–S3). Ph-An-COF consists of ordered 1D channels that are 2.9 nm in diameter and periodic π-columns with benzene vertices and anthracene edges, in which the anthracene units function as photo-responsive building blocks (Figure 1 b–d). Thermogravimetric analysis revealed that Ph-An-COF did not show decomposition up to 400 °C under nitrogen (Figure S2). Ph-An-COF exhibits an X-ray diffraction (XRD) profile with prominent peaks at 3.04, 6.02, 6.72, 8.81 and 26.3°, which are assigned to the 100, 110, 200, 210 and 001 facets, respectively (Figure 1 e, blue curve). The presence of a 001 facet at 26.3° corresponds to a π–π stacking distance of 3.4 Å, indicating that molecular ordering occurs along the direction perpendicular to the 2D planar sheets. Pawley refinement generated an XRD profile (black curve) that reproduced the experimentally observed curve, as evidenced by their negligible difference (dotted black curve). Structural simulations with an eclipsed AA-stacking structure generated an XRD curve (green curve) that is consistent with the experimental profile. By contrast, the staggered AB mode (orange curve) could not reproduce the experimental result. These XRD results indicate that Ph-An-COF is a crystalline material with 1D mesopores and ordered anthracene π-columns (Figure 1 b).

**Figure 1:**
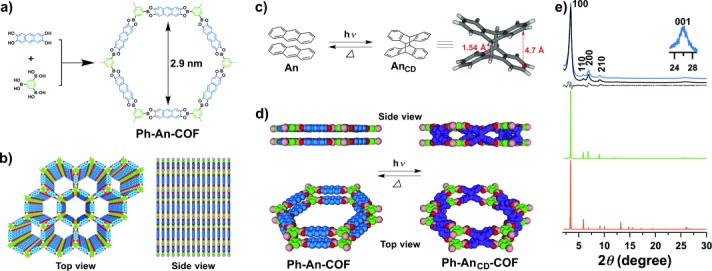
a) Synthesis of anthracene COF (Ph-An-COF). b) Top and side views of Ph-An-COF (blue: anthracene unit). c) [4π+4π] anthracene (An) cycloaddition upon irradiation to form the cyclodimer (AN_CD_) and its thermally allowed reverse reaction (inset: singlet crystal structure of An_CD_). d) Side and top views of the structural changes of one hexagonal macrocycle of the COFs upon photoirradiation and thermal stimulation (blue: anthracene Unit, purple: cycloaddition dimer of anthracene). e) XRD patterns of Ph-An-COF: experimentally observed (blue curve), Pawley refined (black curve), their difference (dotted curve), the patterns simulated using the eclipsed AA (green curve) and staggered AB (orange curve) stacking modes.

The anthracene columns in Ph-An-COF are configured in a face-on-face π-stacking mode with a vertical interlayer separation of 3.4 Å between two anthracene units. Such a stacking mode, together with a suitable distance (<4.2 Å),[8] is favorable for photoinduced [4π+4π] cycloaddition. As shown in Figure 1 c, photoinduced [4π+4π] anthracene cycloaddition is a symmetrically allowed reaction that occurs at the 9- and 10-positions of anthracene (An) and forms concavo-convex-shaped cycloaddition dimers (An_CD_). The cycloaddition results in a decrement of aromaticity because the extended π-system is broken in the dimer (Figure 1 c, inset), whereas a direct single C–C bond of 1.54 Å connecting the two units gives rise to a drastic conformational change, resulting in a shortened distance between the central portions of the dimeric molecules and in expanded spatial separation at both ends (C-to-C distance of 4.7 Å). This cycloaddition reaction is thermally reversible, regenerating two planar anthracene units (Figure 1 c).

With these results in mind, we prepared films of Ph-An-COF by in situ polymerization to deposit a Ph-An-COF film (thickness 242 nm) onto a quartz substrate (Supporting Information). Ph-An-COF exhibited two strong electronic absorption bands at 278 and 373 nm; these bands were assigned to the n–π* and π–π* transition bands of the anthracene units, respectively (Figure 2 a, blue curve). Irradiation of the film under Ar with 360 nm light using a xenon lamp through a band-path filter (360±10 nm) caused the intensity of the absorption bands at 278 and 373 nm to decrease because the cycloaddition reaction disrupted the π-conjugation of the anthracene units.[8] By contrast, the intensity of the band at 305 nm, which was assigned to the n–π* transition band of the cycloaddition dimer, increased. These spectral changes occurred in conjunction with the appearance of clear isosbestic points at 289, 325 and 410 nm, which indicate that the cycloaddition reaction is free of side reactions and proceeds cleanly to form the cycloaddition dimers without any byproducts. We monitored the cycloaddition reaction using time-dependent electronic absorption spectroscopy. A plot of absorbance versus time yielded a linear curve (Figure 2 a, inset); the reaction was first order, with a rate constant of 4.1×10^−3^ min^−1^. All of these spectral changes indicate that Ph-An-COF is responsive to irradiation, which triggers the cycloaddition of the anthracene units in the π-columns, thus transforming the planar 2D sheets into concavo-convex polygon skeletons (Figure 1 d, Ph-An_CD_-COF). We hydrolyzed the photogenerated Ph-An_CD_-COF samples and conducted ^1^H NMR spectroscopy, which confirmed the production of only cyclic anthracene dimers (Figure S4). On the basis of the proton integrations, the photostationary state of the [4π+4π*] cycloaddition was calculated to be 47 % dimers, which is similar to the values of other anthracene systems reported to date.[8]

**Figure 2:**
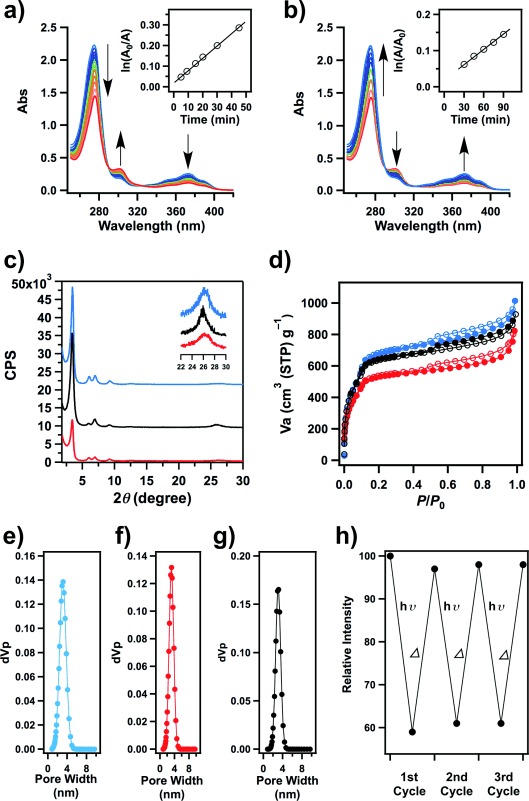
a) Electronic absorption spectral changes of Ph-An-COF upon irradiation at 360 nm (inset: time-dependent profile). b) Electronic absorption spectral changes of Ph-An_CD_-COF upon heating at 100 °C in the dark (inset: time-dependent profile). c) XRD patterns of Ph-An-COF (blue curve), Ph-An_CD_-COF (red curve), and Ph-An-COF (black curve) generated by heating Ph-An_CD_-COF at 100 °C under Ar in the dark. d) Nitrogen sorption isotherm curves (blue: Ph-An-COF; red: Ph-An_CD_-COF; black: Ph-An-COF generated by heating Ph-An_CD_-COF at 100 °C under Ar in the dark; filled circles: adsorption; open circles: desorption). Pores size distribution profiles of e) Ph-An-COF, f) Ph-An_CD_-COF, and g) Ph-An-COF generated by heating Ph-An_CD_-COF. h) Cycle performance as revealed by normalized absorbance change.

The XRD patterns of the photoirradiated COF samples (Figure 2 c, red curve) were similar to that of Ph-An-COF (blue curve), with similar peak positions of the 100, 110, 200, 210 and 001 facets. Photoirradiation stimulated a cycloaddition-induced structural transformation, whereas the crystallinity of the polygon skeletons was retained (Figure 2 c). The observed decrease in the intensity of the XRD peaks is likely to be related to the fact that the concavo-convex polygon skeletons have a relatively flexible conformation, which leads to weak X-ray diffraction.

The alignment of building units along the *c*-axis in Ph-An-COF constitutes 1D mesoporous channels, in which the channel walls may serve as an interface for gas adsorption. The channel walls of the concavo-convex polygon Ph-An_CD_-COF differ from those of the Ph-An-COF. As revealed by nitrogen sorption isotherm measurements, the photoinduced structural transformation is accompanied by a change in gas storage capability. For example, Ph-An-COF exhibits typical type-IV curves, which are characteristic of mesoporous materials (Figure 2 d, blue curve). The Brunauer–Emmett–Teller (BET) and Langmuir surface areas were evaluated to be 1864 and 2782 m^2^ g^−1^, respectively, whereas the pore size and pore volume were calculated to be 2.9 nm and 1.24 cm^3^ g^−1^, respectively (Figure 2 e and S5), using the nonlocal density function theory (NLDFT) method. The photo-generated Ph-An_CD_-COF samples exhibited type-IV sorption profiles (Figure 2 d, red curve) along with BET and Langmuir surface areas of 1456 and 2142 m^2^ g^−1^, respectively. In relation to this observation, the pore volume decreased to 1.08 cm^3^ g^−1^ (Figure S4). Notably, the pore size distribution profile revealed that the photo-generated Ph-An_CD_-COF consists of only one type of mesopore, with a pore size of 2.9 nm (Figure 2 f and S5). This result is reasonable because the irradiation mainly causes the structural transformation along the *c*-axis, whereas the pore diameter is less affected.

The photoinduced structural transformation significantly affects the π-electronic properties of the samples. Ph-An-COF is highly blue-luminescent, exhibiting an emission band centered at 429 nm, as a result of excimer emission of the π-stacked anthracene units (Figure S6). The absolute fluorescence quantum yield of the Ph-An-COF solid samples was evaluated to be 5.4 %. Irradiation of Ph-An-COF caused a monotonic decrease of the fluorescence intensity because the resulting cycloaddition dimers were not luminescent. Time-dependent spectral changes revealed that 42 % of the anthracene units in Ph-An-COF was transformed into dimers in the photostationary state, which is consistent with the value calculated on the basis of the electronic spectral changes.

The results show that the changes of properties, including the porosity, π-electronic absorption and luminescence, are dramatic and correlate with the structural transformation induced by irradiation. Reversibility of such properties is highly desired for wide applications of this type of materials and thus we examined its reversibility. To test the possibility of thermal reversibility, we heated the photo-generated Ph-An_CD_-COF samples at 100 °C in the dark and observed that the dimers returned back to the anthracene units. Electronic absorption spectroscopy confirmed that the cycloaddition dimers were thermally transformed into anthracene units, showing enhanced absorbance at 278 and 373 nm with clear isosbestic points (Figure 2 b). This spectral change also reveals that the thermally induced recovery of anthracene units was nearly quantitative. From the time-dependent profile (Figure 2 b, inset), the reaction was determined to be in first order, with a rate constant of 1.3×10^−3^ min^−1^. Time-dependent fluorescence spectroscopy also suggests that the fluorescence of the anthracene excimer was recovered upon heating (Figure S6). Nitrogen sorption isotherm measurements revealed an enhanced BET surface area of 1684 m^2^ g^−1^ (Figure 2 d, black curve). The pore size and pore volume were evaluated to be 2.9 nm and 1.14 cm^3^ g^−1^, respectively (Figure 2 g and S5). XRD profiles demonstrated that the COF samples recovered the intensity of the peaks while retaining the original diffraction patterns (Figure 2 c, black curve). These observations indicate that thermal stimuli enabled the regeneration of Ph-An-COF. Notably, cycle performance tests revealed that the external-stimuli-responsive transformation is repetitive (Figure 2 h and Figures S7, S8).

The anthracene units stacked in the π-columns of Ph-An-COF are responsive to irradiation, which induces interlayer [4π+4π] cycloaddition reactions, causes conformational changes of the π-columns, and triggers a structural transformation of the layers. These photoinduced hierarchical transformations are reversible by virtue of the thermally allowed reversibility of the cycloaddition reaction. Notably, the structural transformations are accompanied by profound changes in properties and functions, including gas adsorption, π-electronic adsorption and luminescence. Our results demonstrate the first example of photoresponsive structurally dynamic COFs and suggest that COFs could be designed as “smart” materials whose gas adsorption, molecular storage, sensing, and semiconducting properties are controllable by external stimuli.

## Supporting Information

As a service to our authors and readers, this journal provides supporting information supplied by the authors. Such materials are peer reviewed and may be re-organized for online delivery, but are not copy-edited or typeset. Technical support issues arising from supporting information (other than missing files) should be addressed to the authors.

miscellaneous_information
