# Supplementary material for: A Photoresponsive Smart Covalent Organic Framework
Source: Angew Chem Int Ed Engl. 2015 Jun 10;54(30):8704–7. doi: 10.1002/anie.201503902 (PMC4531826; doi:10.1002/anie.201503902)
Supplement: Supplementary file 1 — miscellaneous_information [file anie0054-8704-sd1.pdf]

Angewandte Chemie
Eine Zeitschrift der Gesellschaft Deutscher Chemiker

Supporting Information

A Photoresponsive Smart Covalent Organic Framework**

*Ning Huang, Xuesong Ding, Jangbae Kim, Hyotcherl Ihee, and Donglin Jiang**

anie_201503902_sm_miscellaneous_information.pdf

Contents

Section 1. Methods

Section 2. Materials and synthetic procedures

Section 3. Supporting tables

Section 4. Supporting figures

Section 5. Supporting references

Section 1. Methods

^{1}H NMR spectra were recorded on a JEOL model JNM-LA400 NMR spectrometer, where the chemical shifts (δ in ppm) were determined with a residual proton of the solvent as standard. Fourier transform Infrared (FT IR) spectra were recorded on a JASCO model FT IR-6100 infrared spectrometer. UV-Vis-IR diffuse reflectance spectrum (Kubelka-Munk spectrum) was recorded on a JASCO model V-670 spectrometer equipped with integration sphere model IJN-727. Powder X-ray diffraction (PXRD) data were recorded on a Rigaku model RINT Ultima III diffractometer by depositing powder on glass substrate, from $2\theta = 1.5°$ up to 60° with 0.02° increment. Elemental analysis was performed on a Yanako CHN CORDER MT-6 elemental analyzer. TGA measurements were performed on a Mettler-Toledo model TGA/SDTA851^e under N_2, by heating to 800 °C at a rate of 10 °C min^{-1}. Field-emission scanning electron microscopy (FE-SEM) was performed on a JEOL model JSM-6700 operating at an accelerating voltage of 5.0 kV. The sample was prepared by drop-casting an acetone suspension onto mica substrate and then coated with gold. High-resolution transmission electron microscopy (HR-TEM) images were obtained on a JEOL model JEM-3200. Photoluminescence spectrum was recorded on a JASCO model FP-6600 spectrofluorometer. The absolute quantum yield was determined by standard procedure with an integral sphere JASCO model ILF-533 mounted on the FP-6600 spectrofluorometer. Time-resolved fluorescence spectroscopy was recorded on Hamamatsu compact fluorescence lifetime spectrometer Quantaurus–Tau model C11367-11.

Nitrogen sorption isotherms were measured at 77 K with a micromeritics@ 3Flex model analyzer. Before measurement, the samples were degassed in vacuum at 120 °C for more than 10 h. By using the non-local density functional theory (NLDFT) model, the pore volume was derived from the sorption curve.

Geometry optimization of the unit pore structure was performed at PM3 level by using the Gaussian 03 program package (Revision C.02)[S1] to give the pore size of 2.9 nm in diameter. Molecular modeling and Pawley refinement were carried out using Reflex, a software package for crystal determination from PXRD pattern, implemented in MS modelling ver 4.2 (Accelrys Inc.).[S2] Unit cell dimension was first manually determined from the observed PXRD peak positions by using hexagonal arrangement. We performed Pawley refinement to optimise the lattice parameters iteratively until the R_{WP} value converges. The refinement indicates a

hexagonal crystal system with a unit cell of $a = b = 30.33032$ Å and $c = 3.36227$ Å. The pseudo-Voigt profile function was used for whole profile fitting and Berrar-Baldinozzi function was used for asymmetry correction during the refinement processes. The final R_{WP} and R_P values were 8.73 and 5.98%, respectively. Simulated PXRD patterns were calculated from the refined unit cell and compared with the experimentally observed patterns. This structure could have two distinct arrangements: (1) a staggered AB type arrangement with graphite-like packing, where three-connected vertices lie over the center of the six-membered rings of neighbouring layers; (2) an eclipsed AA type arrangement, where all atoms in an each layer of the framework lie exactly over one another. The AA type arrangement was constructed in space group *P*6/mmm symmetry (space group number 191) and the AB type arrangement was constructed in space group *P*63/mmc symmetry (space group number 194). The atoms are placed on the special position to form the 2D framework where all bond lengths and angles are taken from the optimised geometrical parameters calculated at B3LYP/6-31G(d) to maintain reasonable values. After comparing each simulated pattern with experimentally observed pattern, only the simulated pattern from the eclipsed AA type arrangement shows good agreement with the observed PXRD pattern.

Section 2. Materials and synthetic procedures

Mesitylene, anhydrous dioxane, and anhydrous acetone (99.5%) were purchased from Wako Chemicals. 1,2-dimethoxybenzene and 1,3,5-tribromobenzene were purchased from TCI. 1,3,5-Benzenetriboronic acid was prepared from 1,3,5-tribromobenzene using a literature procedure.[S3] 2,3,6,7-Tetrahydroxyanthracene was prepared from 1,2-dimethoxybenzene using a literature procedure.[S4]

1,3,5-Tris(trimethylsilyl)benzene. A solution of trichlorobenzene (65.0 g, 358 mmol, 98%) in dry THF (200 mL) was added dropwise within 45 min to a mixture of activated magnesium turnings (32.4 g, 1.33 mol) and trimethylsilyl chloride (195 mL, 1.53 mol) in dry THF (200 mL). After stirred at room temperature overnight, the mixture was heated to reflux for 18 h. The mixture was then poured into ice water (250 mL), and the precipitate was collected by a suction filtration. The residue was extracted with diethyl ether (2 × 50 mL) and the combined organic layers were washed with saturated aqueous NH_4Cl (100 mL) and water, and then dried over anhydrous $MgSO_4$. After filtration, the solvent was evaporated and the crude product was purified by distillation under reduced pressure (bp = 91-98 °C at 1.5 mmHg) to give a colorless liquid (74 g, 70%). ^1H NMR (400 MHz, $CDCl_3$, 25 °C): δ (ppm) 7.72 (s, 3H, Ar-H), 0.31 (s, 27H, $Si(CH_3)_3$). ^{13}C NMR (125 MHz, $CDCl_3$, 25 °C): δ (ppm) 138.82, 138.29, -1.04.

1,3,5-Benzenetriboronic acid. 1,3,5-Tris(trimethylsilyl)-benzene (9.82 g, 33.4 mmol) was treated with neat boron tribromide (41.0 g, 0.164 mol) under argon. A condenser was attached that was also charged with argon, and the solution was heated at 100 °C for 4 h. Once cooled, excess boron tribromide was distilled under vacuum (1 Torr) at room temperature. The resulting gray-purple solid was dissolved in dry hexane (50 mL) and cooled to 0 °C with an ice bath. Water was slowly added dropwise while stirring vigorously until the reaction had been fully quenched. The gray solid was collected by filtration and rinsed with water. After drying under vacuum (1 Torr) for 72 h, the product was obtained as the trihydrate in >99% yield. ^1H NMR (400 MHz, 1 M KOH in D_2O): δ (ppm) 7.33 (s, 3 H).

2,3,6,7-Tetramethoxy-9,10-anthraquinone. 9,10-Diethyl-2,3,6,7-tetramethoxyanthracene (737 mg, 2.08 mmol) and $K_2Cr_2O_7$ (3.06g, 10.4 mmol) were suspended in 25 mL of acetic acid and then heated to 90 °C for 30 minutes. After the mixture was cooled to room temperature, the resulting yellow solid was filtered, washed with water to remove the unreacted $K_2Cr_2O_7$, and

finally washed with Et_2O. The solid was dried in the air to yield 2,3,6,7-tetramethoxy-9,10-anthraquinone (270 mg) as a yellow crystalline solid in 40% yield. ^1H NMR (400 M, $CDCl_3$): δ (ppm) 4.07 (s, 12H), 7.69 (s, 4H).

2,3,6,7-Tetramethoxy-9(10H)-anthracenone. 2,3,6,7-Tetramethoxy-9,10-anthraquinone (352 mg, 1.07 mmol) was mixed with 10% Pd/C (50 mg) in 40 ml of acetic acid at 90 °C for 45 minutes under H_2 atmosphere. The reaction mixture was concentrated under reduced pressure and extracted with $CHCl_3$. The resulting solution was washed with 10% $NaHCO_3$, brine, and then dried with anhydrous $MgSO_4$. Filtration and removal of the solvent under reduced pressure yielded a light yellow powder (299 mg). ^1H NMR (400 MHz, $CDCl_3$): δ (ppm) 3.96 (s, 6H), 3.99 (s, 6H), 4.06 (s, 2H), 6.76 (s, 2H), 7.75 (s, 2H).

2,3,6,7-Tetramethoxyanthracene. 2,3,6,7-Tetramethoxy-9(10H)-anthracenone (409 mg, 1.30 mmol) and $LiAlH_4$ (247 mg, 6.50 mmol) in anhydrous THF (45 ml) were stirred under argon atmosphere at room temperature for 30 minutes. The reaction was quenched with ethyl acetate, and the solvent was evaporated under reduced pressure. To the resulting residue was added H_2O and concentrated HCl to achieve a $pH < 1$. This suspension was then extracted twice with $CHCl_3$. The organic layers were washed with 10% $NaHCO_3$ solution, water and brine, and then dried with anhydrous $MgSO_4$. After filtration and removal of the solvent, the residue was subject to silica chromatography with CH_2Cl_2 as eluent to yield 2,3,6,7-tetramethoxyanthracene as white crystalline solid (277 mg). ^1H NMR (400 MHz, $CDCl_3$): δ (ppm) 4.02 (s, 12H), 7.12 (s, 4H), 8.01 (s, 2H).

2,3,6,7-Tetrahydroxyanthracene. To 2,3,6,7-Tetramethoxyanthracene (267 mg, 0.90 mmol) suspended in anhydrous CH2Cl2 (50 ml) at 0 °C under a nitrogen atmosphere was added BBr3 (0.6 ml, 0.3 mmol) drop-wise. The reaction mixture was heated to reflux in argon atmosphere for 2 h, and then poured into cold 0.1 M HCl (150 ml) with stirring. The resulted white precipitate was extracted with diethyl ether (three times). The ether solution was washed with water, brine and dried over $MgSO_4$. Filtration and removal of the solvent under reduced pressure yielded light brown powder (210 mg) that was used without further purification. ^1H NMR (400 MHz, acetone-d^6): δ (ppm) 7.20 (s, 4H), 7.89 (s, 2H), 8.45 (s, 4H).

Ph-An-COF. A mixture of 1,3,5-benzenetriboronic acid (21 mg, 0.1 mmol) and 2,3,6,7-tetrahydroxyanthracene (36.3 mg, 0.15 mmol) in 1,4 dioxane/mesitylene (2 mL, 1/1 in vol.) in a 10 mL Pyrex tube was degassed through three freeze-pump-thaw cycles. The tube was

sealed and heated at 120 °C for 3 days. The resulting precipitate was collected by centrifugation, washed with anhydrous acetone, and dried at 150 °C under vacuum to produce Ph-An-COF (16.3 mg) in 92% isolated yield as a pale yellow powder.

Preparation of Ph-An-COF thin film on quartz substrates. Before use, the quartz substrate was washed by boiling water, acetone and boiling isopropanol and then dried under vacuum at 120 °C for 10 h. A mixture of 1,3,5-benzenetriboronic acid (3.5 mg, 0.1 mmol) and 2,3,6,7-tetrahydroxyanthracene (6.1 mg, 0.15 mmol) in 1,4 dioxane/mesitylene (25 mL, 1/1 in vol.) in a 50 mL Schlenk tube was degassed through three freeze-pump-thaw cycles. After that, the washed quartz substrate (8 mm × 8mm) was carefully put in the tube. The tube was sealed and heated at 120 °C for 3 days. Cooling to room temperature, the glass substrate was taken out, washed with anhydrous acetone, and dried under vacuum to yield Ph-An-COF as a pale yellow thin film on quartz. A photo of a typical film is shown below and the film thickness is 242 nm as measured by using profilometer (Veeco Dektak 6M).

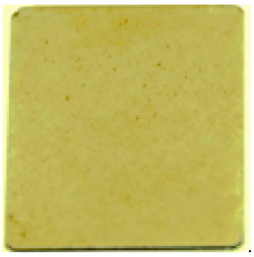

A Photo of COF film (1 X 1 cm)

Photoirradiation and thermal treatments. Light irradiation experiments were conducted under Ar by using light at 360 nm through band-path filter of a xenon lamp at 22 °C. At designated period of photoirradiation, the films were monitored using electronic absorption and fluorescence spectroscopy. For the reverse thermal induced reactions, the films after photoirradiation were heated at 100 °C under Ar in the dark and monitored using electronic absorption and fluorescence spectroscopy. To evaluate the stimuli-induced porosity change, powder samples were utilized. The Ph-An COF powder was irradiated with the xenon lamp under Ar, and the resulting Ph-An_{CD} COF powder was degassed at room temperature under high vacuum and subjected to nitrogen adsorption measurements at 77 K. For thermal reversibility study, the Ph-An_{CD} COF powder samples were heated at 100 °C under Ar in the dark and subjected to nitrogen adsorption measurements at 77 K.

Section 3. Supporting tables

Table S1. Elemental analysis results of Ph-An-COF

COFs		C%	H%	N%
Ph-An-COF	Calcd.	76.95	4.25	11.97
	Found	74.92	4.88	10.87

Table S2. IR spectral assignment of Ph-An-COF

**Peak (cm^{-1})**	**Assignment and Notes**
3420 (m)	O–H stretch from the end $B(OH)_2$ or OH groups
3037 (w)	Aromatic C–H stretch from benzene units
2923 (w)	C–H stretching from anthracene building blocks
1594 (m)	C=C stretch for phenyl rings.
1456 (m)	C=C vibrational modes for anthracene building blocks
1326 (m)	B–O stretch, characteristic band for boroxoles
1308 (s)	B–O stretch
1206 (s)	C–O stretch, characteristic for boroxoles
1105 (w)	C–H in-plane bending mode
897 (m)	C–H out-of-plane bending modes
784 (w)	C–H out-of-plane bending modes
704 (w)	

Table S3. Lattice parameters from Pawley refinement

Formula	$C_{54}O_{12}B_6$
Formula weight	905.44
Crystal system	Hexagonal
Space group	*P*6/mmm (No.191)
Unit cell dimensions	$a = b = 30.33302$ Å<br>$c = 3.36227$ Å<br>$\alpha = \beta = 90°$, $\gamma = 120°$
Cell volume	2678.66 $Å^3$
Density calculated	0.601505 g cm^{-3}

Table S4. Atomic coordinates of Ph-An-COF

**Atom**	**x**	**y**	**z**	**g**	**u**	**Wyck**
1 B	0.21964	0.60947	0.50000	1.000	1.000	12q
2 O	0.18960	0.55612	0.50000	1.000	1.000	12q
3 C	0.13934	0.54594	0.50000	1.000	1.000	12q
4 C	0.09536	0.49902	0.50000	1.000	1.000	12q
5 C	0.04736	0.49956	0.50000	1.000	1.000	12q
6 C	0.27892	0.63946	0.50000	1.000	1.000	6m
7 C	0.30677	0.69323	0.50000	1.000	1.000	6m
8 C	0.54631	0.54631	0.50000	1.000	1.000	6k

Section 4. Supporting figures

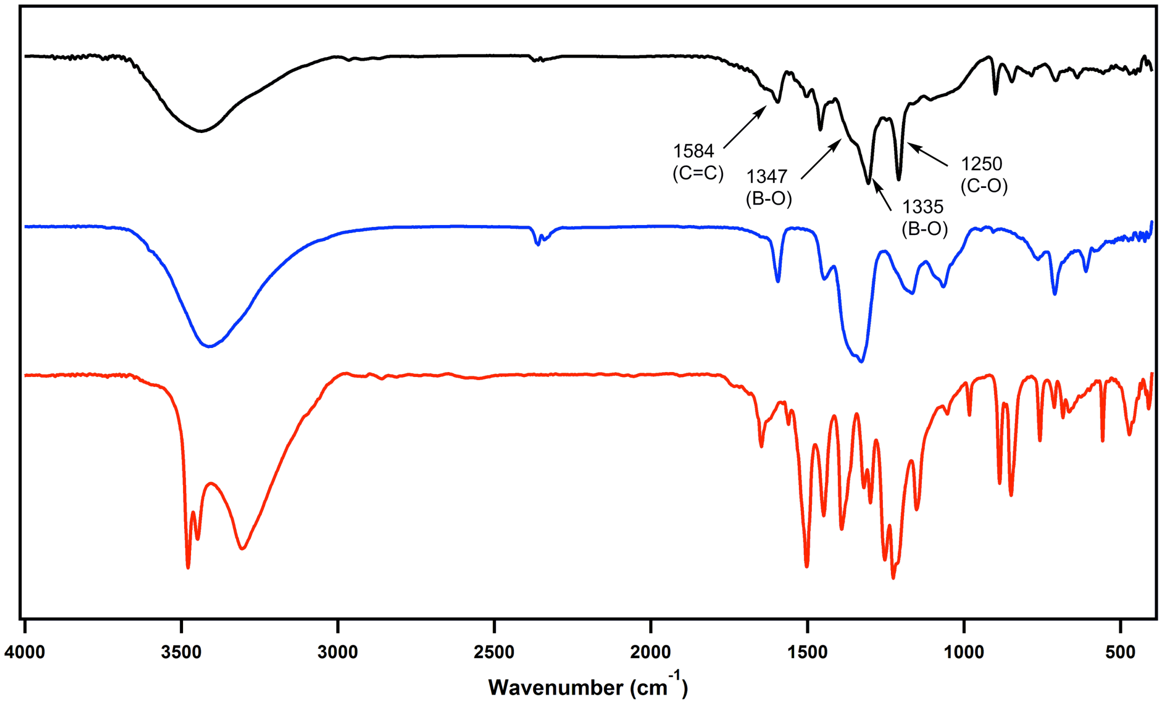

Figure S1. FT IR spectra of Ph-An-COF (black curve), 1,3,5-benzenetriboronic acid (blue curve), and 2,3,6,7-tetrahydroxyanthracene (red curve).

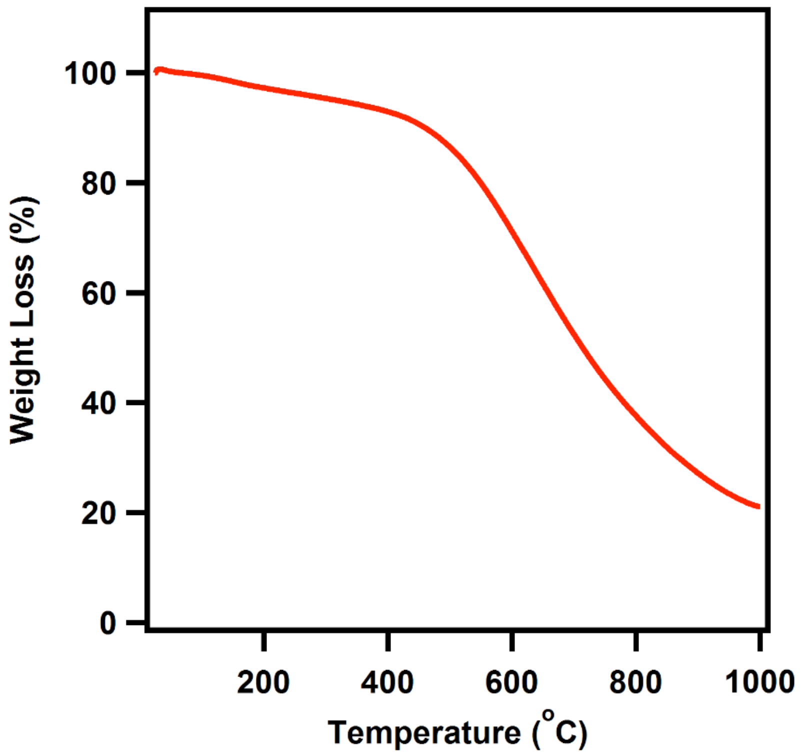

Figure S2. TGA curve of Ph-An-COF.

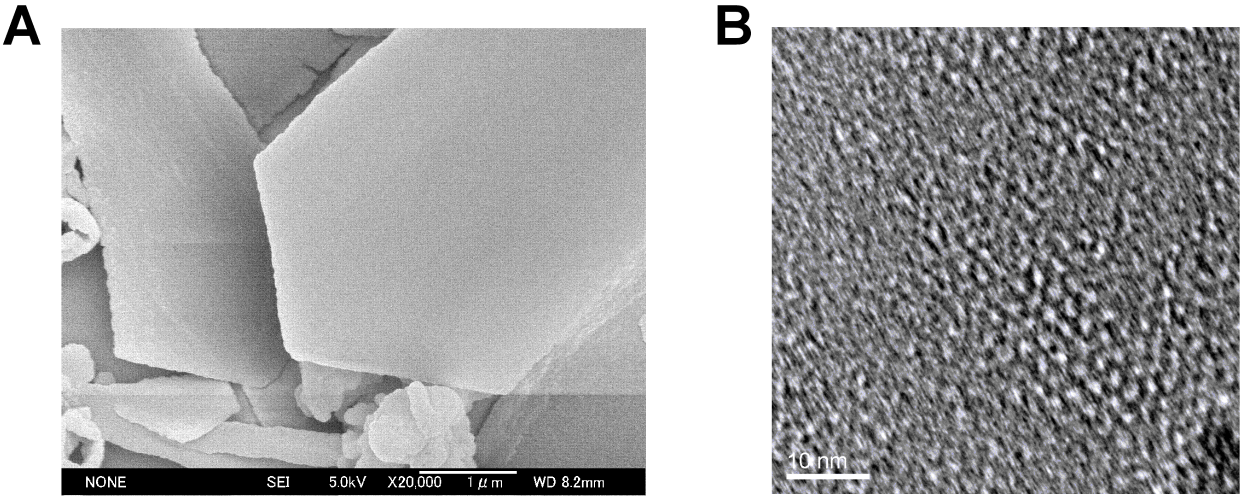

Figure S3. (A) FE-SEM and (B) HR-TEM images of Ph-AN-COF.

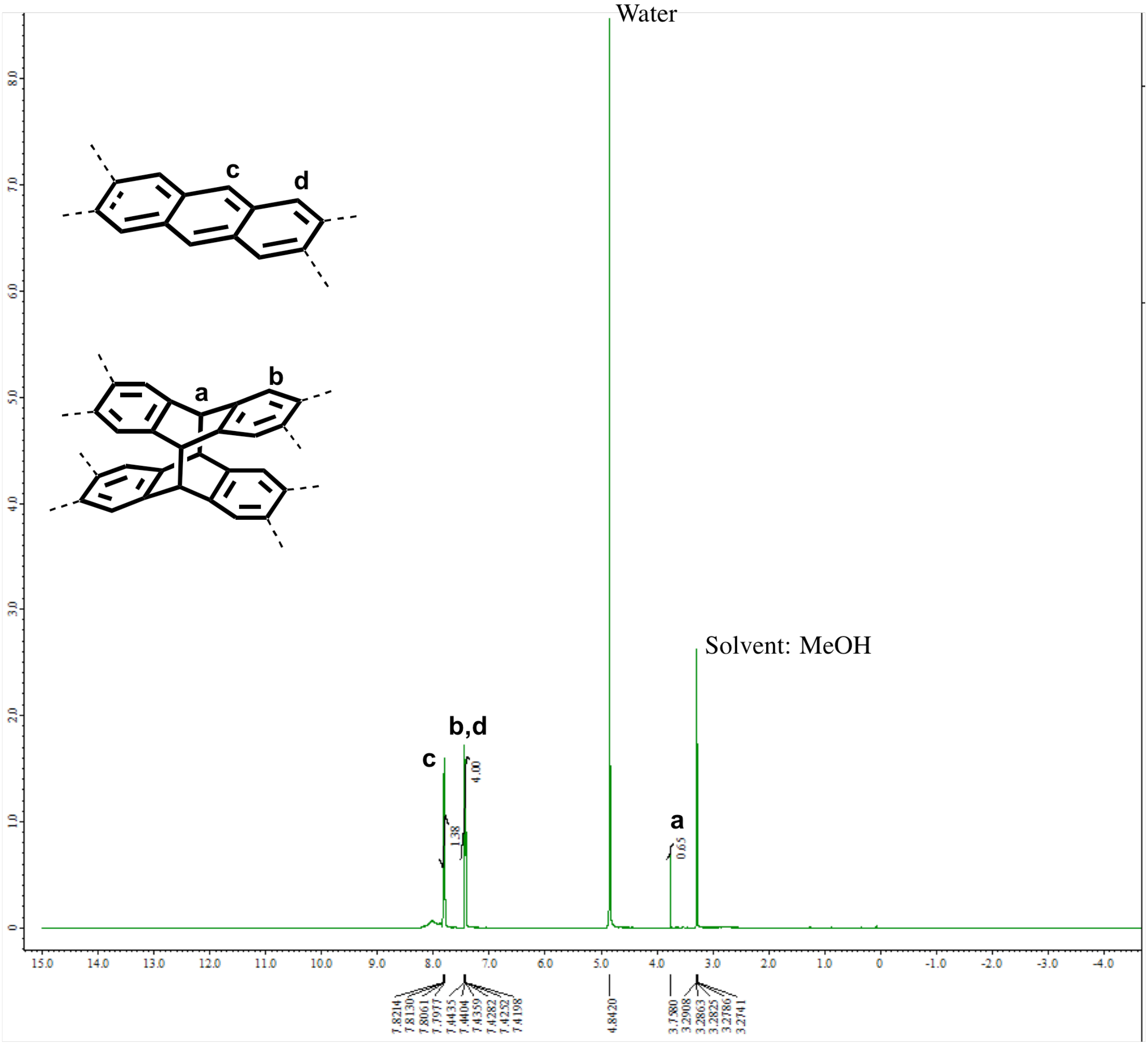

Figure S4. ^{1}H NMR spectrum of hydrolyzed Ph-An$_{CD}$-COF in d_4-MeOD. From the integration of proton, the photogenerated dimer is 47%.

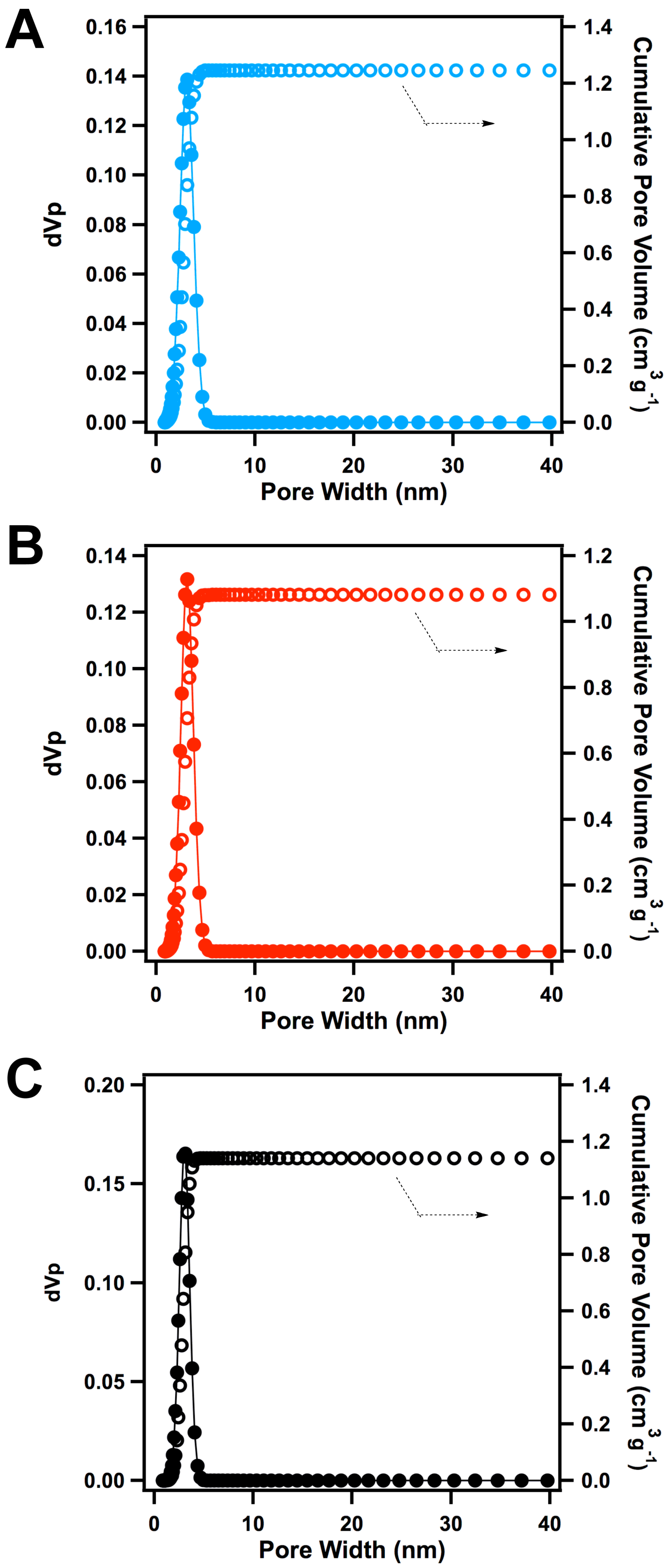

Figure S5. Pore size and pore size distribution profiles of (A) Ph-An-COF, (B) Ph-An$_{CD}$-COF photo-generated and (C) Ph-An-COF upon heating Ph-An$_{CD}$-COF at 100 °C in the dark.

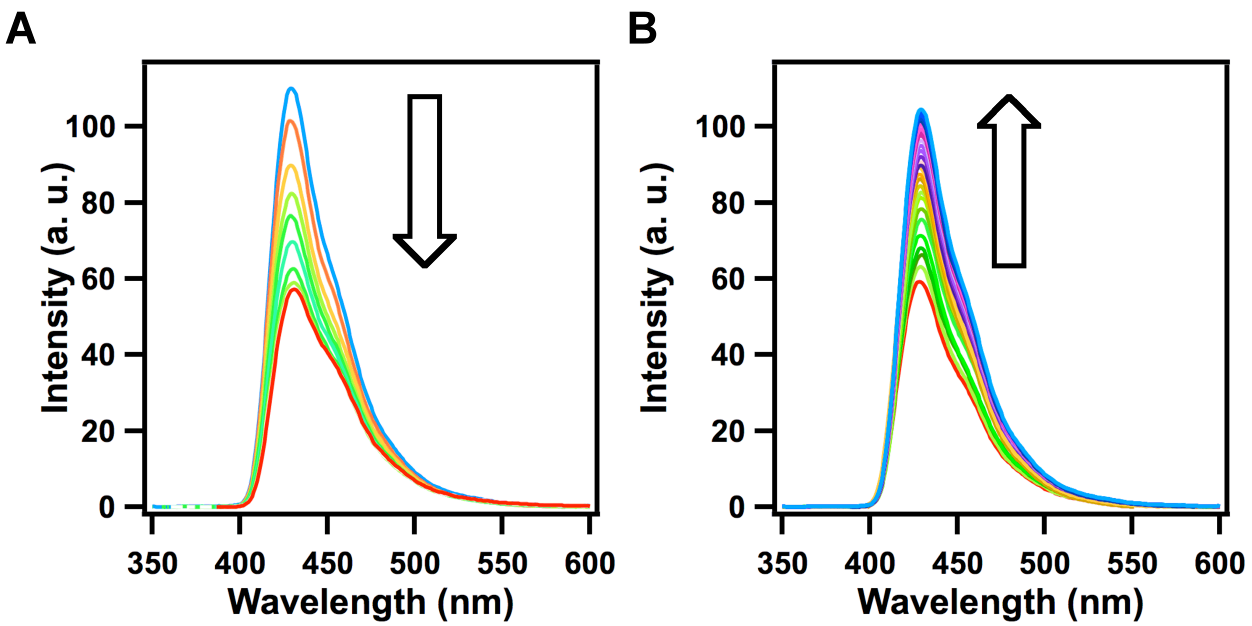

Figure S6. Fluorescence spectral changes of (A) Ph-An-COF upon photoirradiation under Ar and (B) photoirradiated COF upon heating at 100 °C in the dark.

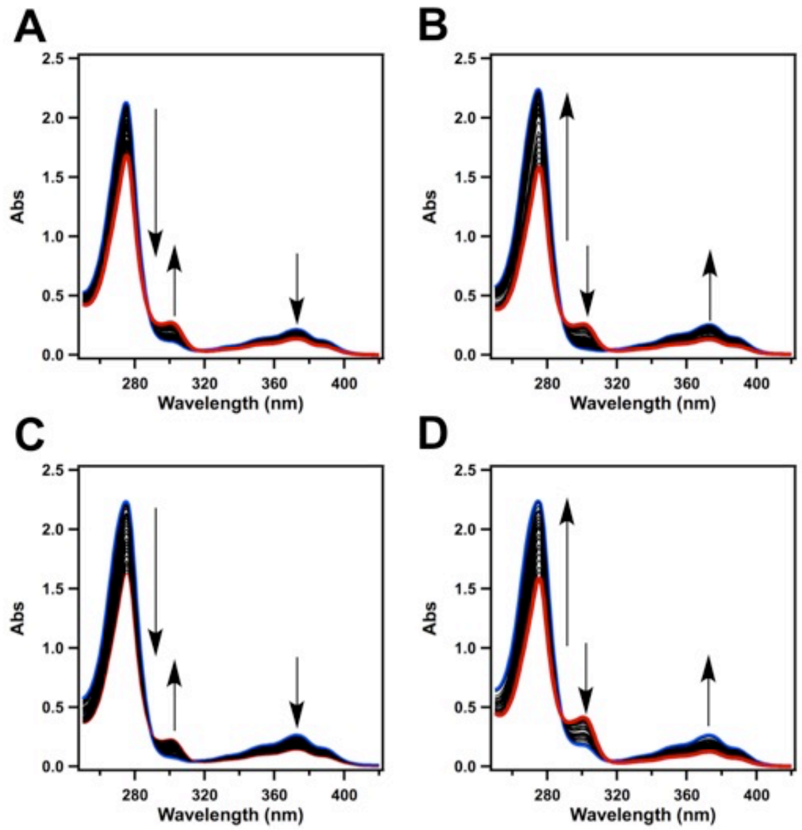

Figure S7. Cycle test as monitored by electronic spectral changes of (A) 2nd photoirradiation and (B) 2nd thermal stimulus, (C) 3rd photoirradiation and (D) 3rd thermal stimulus. The spectral changes upon 1st photoirradiation and thermal stimulus were presented in Figure 2.

For the sample of Ph-An-COF

BET constant C = 55.3, Correlation coefficient = 0.999, S_{BET} = 1864 m^2 g^{-1}

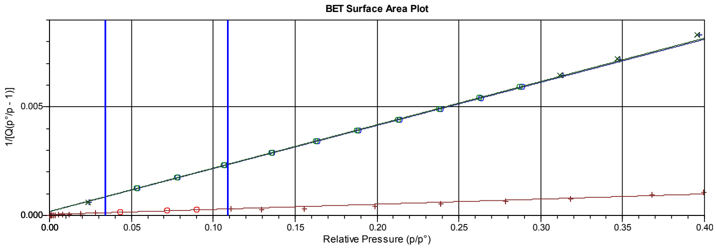

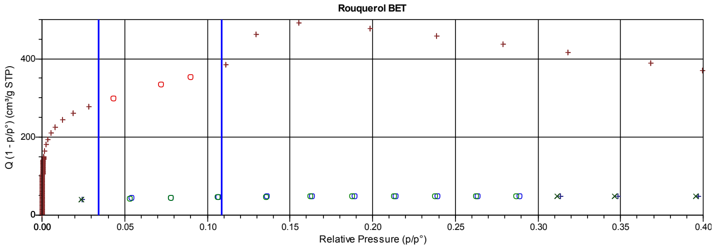

For the sample of Ph-An$_{CD}$-COF

BET constant C = 25.9, Correlation coefficient = 0.999, S_{BET} = 1456 m^2 g^{-1}

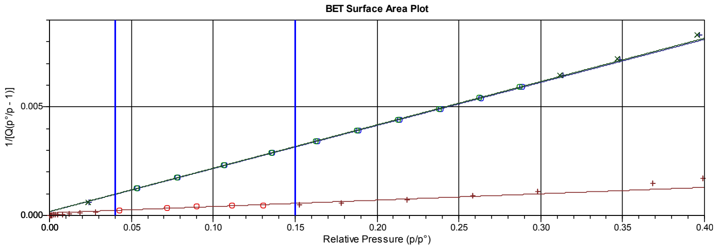

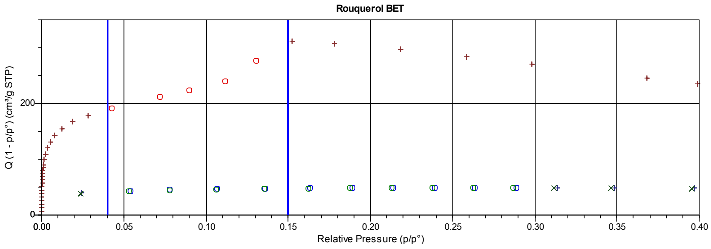

For the sample of Ph-An-COF generated by heating Ph-An$_{CD}$-COF

BET constant C = 33.6, Correlation coefficient = 0.999, S_{BET} = 1684 m^2 g^{-1}

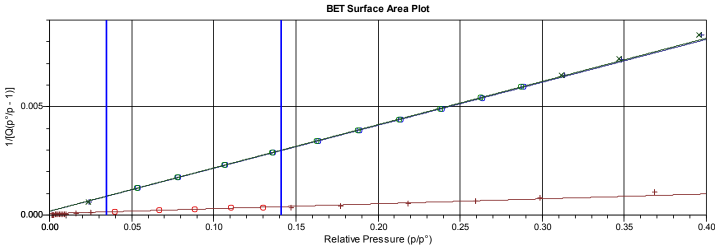

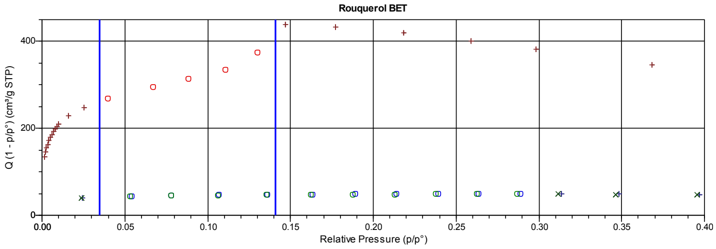

Figure S8. Detailed data for the gas sorption measurements including BET plots, BET constant C, and Rouquerol plots.

Section 5. Supporting references

S1. Gaussian 03, Revision C.02, Frisch, M. J.; Trucks, G. W.; Schlegel, H. B.; Scuseria, G. E.; Robb, M. A.; Cheeseman, J. R.; Montgomery, Jr., J. A.; Vreven, T.; Kudin, K. N.; Burant, J. C.; Millam, J. M.; Iyengar, S. S.; Tomasi, J.; Barone, V.; Mennucci, B.; Cossi, M.; Scalmani, G.; Rega, N.; Petersson, G. A.; Nakatsuji, H.; Hada, M.; Ehara, M.; Toyota, K.; Fukuda, R.; Hasegawa, J.; Ishida, M.; Nakajima, T.; Honda, Y.; Kitao, O.; Nakai, H.; Klene, M.; Li, X.; Knox, J. E.; Hratchian, H. P.; Cross, J. B.; Bakken, V.; Adamo, C.; Jaramillo, J.; Gomperts, R.; Stratmann, R. E.; Yazyev, O.; Austin, A. J.; Cammi, R.; Pomelli, C.; Ochterski, J. W.; Ayala, P. Y.; Morokuma, K.; Voth, G. A.; Salvador, P.; Dannenberg, J. J.; Zakrzewski, V. G.; Dapprich, S.; Daniels, A. D.; Strain, M. C.; Farkas, O.; Malick, D. K.; Rabuck, A. D.; Raghavachari, K.; Foresman, J. B.; Ortiz, J. V.; Cui, Q.; Baboul, A. G.; Clifford, S.; Cioslowski, J.; Stefanov, B. B.; Liu, G.; Liashenko, A.; Piskorz, P.; Komaromi, I.; Martin, R. L.; Fox, D. J.; Keith, T.; Al-Laham, M. A.; Peng, C. Y.; Nanayakkara, A.; Challacombe, M.; Gill, P. M. W.; Johnson, B.; Chen, W.; Wong, M. W.; Gonzalez, C.; and Pople, J. A.; Gaussian, Inc., Wallingford CT, 2004.

S2. Accelrys, Material Studio Release Notes, Release 4.2, Accelrys Software, San Diego 2006.

S3. R. William Tilford , William R. Gemmill , Hans-Conrad zur Loye , and John J. Lavigne, *Chem. Mater.* **2006**, *18*, 5296–5301.

S4. Jonas Hellberg, Emma Dahlstedt, Margit E. Pelcman, *Tetrahedron* **2004**, *60*, 8899-8912.
